# Ancient Legacy of Cranial Surgery

**DOI:** 10.5812/atr.6556

**Published:** 2012-08-21

**Authors:** Mohammad Ghannaee Arani, Esmaeil Fakharian, Fahimeh Sarbandi

**Affiliations:** 1 Trauma Research Center, Kashan University of Medical Sciences, Kashan, IR Iran

**Keywords:** Wound and Injuries, Ancient History, Trephining

## Abstract

Cranial injury, as it is known today, is not a new concern of modern medicine. On stepping on the earth, the man was in reality encountered with various types of injuries, particularly those of a cranial nature. Leading a life, whether wild or civilized, has always been associated with injuries for human race from the very beginning of birth. Therefore, managing cases of this type has gradually forced him to establish and fix strategies and approaches to handle the dilemma. This study is thus focused on tracing the first documented traumatized cranial cases ever reported, ranging from those trials attributed to our ancient predecessors to the identical examples in the present time.

## 1. Introduction

### 1.1. "Where is the Wisdom we Have Lost in Knowledge? Where is the Knowledge we Have Lost in Information?" (1)

The history of brain surgery dating to the Neolithic Age was often accompanied with trepanation. Trepanation is defined as a procedure in which a hole is created through the skull, sometimes with a stone tool. Since early times, the terms trepanation and trephination turned up to be used interchangeably. The Greek word "Trypanon," from which the word trepanation was derived means a border and the operation was undertaken in the Neolithic times "where the head was kept still by a frame or brace in the manner of the carpenter's wimple." Trephination, deriving from French origin on the other hand, is meant involvement of a cutting instrument rotating around a center. What is inferred from whatever has been already discussed is that in both methods, a piece of bone is removed from the skull with a saw-like instrument, implying that they are doing more than making a perforation in the calvarium (2).

## 2. Main Text

Officially speaking, trepanation discovery dates back to Paul Broca, Victor Horsley, and the era of Hippocrates, but some archeological findings have revealed skulls that were trepanned thousands of years BC all around the world. The Edwin Smith Papyrus (17th century BC) is known as the oldest written document in which different types of head injuries were reported from a medical viewpoint. This treatise consists of 48 cases, of which 27 ones are related to head trauma, which all contributed to skull fractures (3).

### 2.1. Trepanation: Military, Medical, and Cultural

According to the French physician Paul Broca, ancient physicians were quite familiar with trepanation in which a hole was made in the skull by cutting or drilling it. They did so to alleviate pressure on the brain following an injury to the head, or to release evil spirits from the heads of mentally ill people (4). Cranial trepanation was first recorded by Hippocrates (460–355 BC). A Hippocratic corpus, Celsus (with his famous explanation for how the trepan could be rotated by a bow string around the cylinder) (5), Heliodorus, and Galen all suggested trepanation for splintered fractures of the cranial vault and for closed head traumas (6). Celsus, in particular, devised a method of his own that differed from that of prehistoric times. He advises trepanation for head wounds and gives careful and precise instructions on methodology in his treaties De Medicina, and part of his De Artibus, written between 25 and 35 AD (7). The main concern of Greek physicians was about humors seeping into the skull which led them to the conviction that it was better for the wounds in the head to be left without a bandage. Thus, Galen believed that pressure on a bone could not help to wring out its bad humors and might even push them inward, so it was better to let them ooze out freely (8).

Evidence from the early times shows that man has endeavored to protect himself against injuries and traumas jeopardizing the head in particular. From twenty centuries before Christ, warriors in Egypt wore some form of head protectors. Documentations reveal that not only Pharaoh Thutmose III wore a golden helmet into battle, but two hundred years later, Egyptian mercenaries also used brass head protection. It seems that the honor of making helmets from bronze and adding some extensions to them to protect more parts of the face and head goes to the Assyrians, Babylonians, and ancient Greeks, so that by 500 BC the entire head and face were covered, leaving only small holes for vision and ventilation (2). The German military doctor, Frolich describes cases of trauma between the Greeks and the Trojans during the battle of Troy. He counts 31 head injuries (21%), 16 neck injuries (11%), 79 injuries of the trunk (54%), 10 wounds of the upper extremity (7%), and 11 wounds of the lower extremity (7%). He concluded that in total 114 injuries were fatal (77.5%), while those with head injuries died. Only 14% of the cases with their extremities involved were fatal (9). Pursuing the surgical teachings and techniques of Paulus Aegineta, the Moslem physician Abu al-Qasim al-Zahrawi (called in English Albucasis, 936-1013 AD), performed trepanation using a drill that was not to penetrate the brain. His description of the operation is as follows: “You cut through the bone in the confident knowledge that nothing inward can happen to the membrane even though the operator can be the most ignorant and cowardly of men; yes even if he be sleepy,” but if the dura turned black, “you may know that he is doomed” (10). Bernard de Montfaucon, found the first specimen of a trepanned skull in 1685 in Cocherel, France. Following this discovery, 120 other prehistoric skulls dating back to 6 500 BC were later found in France, of which 40 skulls had holes made by trepanation. Later, evidence for skulls trepanned for military, medical, or cultural purposes turned up one after the other in Germany, Portugal, Italy, and Austria. A trepanated skull belonging to an 11 or 12 year old child was found in Iran in 1966 dated 1100 – 800 BC in the Iron Age period. More recently, another Iranian skull, 51 cm in circumference belonging to a girl aged 13 or 14 years old, was discovered in Shahr-e Sokhta, the “Burned City” (3200 –2000 BC) dating back to 2800 BC. Peru has been also notorious for and home to the largest samples of prehistoric trepanned skulls practiced over a long period of time and across a broad geographical area. According to researchers, the earliest Peruvian trepanations may have been performed 400 BC on the southern coast of Peru circa (11). Archeologists have proven that cranial operations performed using different techniques have a history of at least 7000 years. The majority of cases are assumed to have been carried out for magical purposes so as to drive evil spirits out of the head. Nevertheless, ancient medical literature suggests that medical doctors also observed the injured carefully and consequently, they possessed a good knowledge of severe head injuries which led to a number of accurate medical diagnoses. 

## 3. Discussion

Persian physicians in the Middle Ages such as, Rhazes and Avicenna along with other Muslim physicians like Avenzoar and Albucasis, contributed much to our knowledge of the technical concepts of trephination. However, it was the pioneer of neurosurgery, Albucasis (936-1 013 CE) who gave us a more precise description of many aspects of neurosurgical pathology, its treatment, instruments, and neurosurgical techniques in his treatise. Concepts such as incision in the skin in a cross shape, trephination of the bone outside the suture, trying to keep the dura mater intact, and removal of fragments of broken bones were all documented in a systematic trepanation procedure introduced by Islamic physicians. Albucasis’ book “Kitab Al Tasrif Li-man Ajiza an al-Talif” (Presentation to Would-be Authors of Medicine) translated into Latin, Hebrew, and French was the basis for teaching medicine, especially surgery, in many medical schools in Europe during the Renaissance period. He also introduced the first surgical instruments in his monograph, which included 190 diagrams of surgical instruments, most of which are precursors of those used nowadays (12). He dealt with a multitude of topics concerning neurosurgical pathology such as head injuries, spinal fractures, tumors, hydrocephalus, and functional surgery. Cranial surgery has ever been a highly specialized subject. All writers on the subject, from Hippocrates onwards, therefore mention or describe an interesting range of special instruments. The prince of physicians, Avicenna, described a number of instruments for managing situations where the cranium was concerned. According to him, some instruments are required to perforate the bone, some for pulling out the bones, and some for elevating depressed bones. All of the tools have different shapes due to the diversity of human heads and situations faced by the physician. Thus, the prudent physician is always expected to have his instruments ready, in a range of shapes and in a great number, so that he can if necessary extract, cut, elevate, saw, scrape, or grind the bones in order to achieve a successful outcome. Avicenna described an instrument, a drill calling it a trepanum or a perforator which did not go too deeply into the interior of the surface of the bone. It was so called, because it did not penetrate the dura membrane since it had a round blunt extremity and a little ring which prevented the drill bit from going too deeply into the skull (13). Trephining is still practiced in modern neurosurgical medicine, not only as an important procedure, but beyond its therapeutic characteristics it is also used for; exploratory diagnosis, for relieving intracranial pressure (as from an epidural or subdural hematoma), for debridement of a penetrating wound, and to gain access to the dura and thus to the brain itself to provide for instance, a port through which a stereotactic probe can be introduced into the brain (14). What is obvious and indisputable regarding the flourishing of cranial surgery through medical history is that if early practitioners, especially those of the Greek origin, had not initially become proficient in seeking cranial remedies, we would not have such systematic and safe processes for handling cranial cases as we do today. Undoubtedly, the role of Moslem physicians such as Albucasis, Avicenna, etc, was also decisive in the implementation of the endeavors started by the ancients.
